# Treatment Modalities and Survival Outcomes in Gastric Cancer: Insights From Najran, Saudi Arabia

**DOI:** 10.7759/cureus.79649

**Published:** 2025-02-25

**Authors:** Ahmed M Badheeb, Ibrahim A Alyami, Ahlam Y Alyami, Mohammed Alyami, Mugahed Al Walani, Samer Alkarak, Abdelaziz A Aman, Fahad M Albaiji, Ali G Al Masad, Abdullah S Alyami, Islam A Seada, Abdullah Abu Bakar

**Affiliations:** 1 Oncology, King Khalid Hospital, Najran, SAU; 2 Medicine, Hadhramout University, Mukalla, YEM; 3 Internal Medicine, King Khalid Hospital, Najran, SAU; 4 Medicine, Najran University, Najran, SAU; 5 Colorectal and Oncology Surgery, King Khalid Hospital, Najran, SAU; 6 Gastroenterology and Advanced Endoscopy, King Khalid Hospital, Najran, SAU; 7 General Surgery, King Khalid Hospital, Najran, SAU; 8 Internal Medicine/Endocrine and Diabetes, King Khalid Hospital, Najran, SAU; 9 Gastroenterology and Hepatology, King Khalid Hospital, Najran, SAU; 10 Cardiothoracic Surgery, King Khalid Hospital, Najran, SAU; 11 Ophthalmology, King Khalid Hospital, Najran, SAU

**Keywords:** gastric cancer, mortality, najran, saudi arabia, survival

## Abstract

Background: Gastric cancer (GC) is the fifth most prevalent cancer worldwide and the fourth leading cause of cancer-related mortality. The survival of GC patients is significantly affected by the timing of diagnosis, as late-stage detection drastically reduces survival rates. This study investigates treatment modalities, survival outcomes, and mortality factors of GC patients in Najran, Saudi Arabia.

Materials and methods: A retrospective analysis was conducted involving 121 patients diagnosed with GC treated at King Khaled Hospital in Najran from January 1, 2014, to December 31, 2022. Clinical and pathological parameters, treatment interventions, and survival outcomes were extracted and analyzed from medical records. The Kaplan-Meier method created survival curves and assessed survival probabilities over time. Univariate and multivariate Cox regression analyses identified independent factors associated with mortality risk, calculating hazard ratios (HR) and 95% confidence intervals (CI) to evaluate associations.

Results: The median age of the patients was 64 years, with 70 (57.9%) being over 60 years old. The cohort included 86 males (71.1%) and 35 females (28.9%), resulting in a male-to-female ratio of 2.5:1. Notably, 24 patients (19.8%) were underweight, 49 patients (40.5%) had hypertension, and 33 patients (27.3%) had diabetes. *Helicobacter pylori* infection was present in 18 patients (14.9%). Human epidermal growth factor receptor-2 (HER2) status was negative in 38 patients (31.4%). Elevated levels of carcinoembryonic antigen (CEA ≥ 5 ng/ml) and CA19-9 (≥ 37 U/mL) were noted in 51 patients (42.1%) and 50 patients (41.3%), respectively. Surgical intervention was performed on 72 patients (59.5%), while 49 patients (40.5%) were deemed non-resectable. Among the surgical cohort, neoadjuvant therapy was administered to 36 patients (30%), while others underwent initial or staged surgeries. Pathological findings predominantly showed 95 cases (78.5%) of intestinal-type adenocarcinomas compared to 26 cases (21.5%) of diffuse type. The most common TNM (tumor, node, metastasis) stage was IV (50 patients, 41.3%), followed by stage III (25 patients, 20.7%). During a follow-up of 41.3 ± 38.8 months, 96 patients (79.3%) were alive, while 25 patients (20.7%) had died. The median survival time was 28 months (95% CI: 20-51 months). The one-year, three-year, and five-year survival rates were 68.2% (82 patients), 42.8% (52 patients), and 37.4% (45 patients), respectively. Increased mortality was significantly associated with female gender (HR: 3.12; 95% CI: 1.56-6.24; p = 0.0013), stage IV disease (HR: 1.92; 95% CI: 1.01-3.66; p = 0.0481), and elevated CEA levels (HR: 1.95; 95% CI: 1.08-3.53; p = 0.0277). In contrast, neoadjuvant chemotherapy was linked to reduced mortality (HR: 0.55; 95% CI: 0.31-0.97; p = 0.0374).

Conclusions: The findings of this study indicate a five-year survival rate of 37.4% (95% CI: 29.3-47.8%) among GC patients in Najran. Notably, female gender, advanced disease stage, and elevated CEA levels emerged as significant predictors of increased mortality. Conversely, the administration of neoadjuvant chemotherapy was associated with a reduced mortality risk. These results emphasize the critical need for personalized treatment strategies and robust risk assessments to improve patient outcomes, particularly high-risk ones.

## Introduction

Gastric cancer (GC) is a global health challenge that has been recognized as the fifth most prevalent malignancy in terms of incidence and the fourth in cancer-related mortality [1]. According to the World Health Organization, GC accounts for 5.6% of all cancer cases and 7.7% of cancer-related deaths, respectively [1]. In Saudi Arabia, the situation is equally concerning, with GC representing 2.4% of the total cancer burden and responsible for 4% of cancer fatalities, with a five-year prevalence of 3.21 per 100,000 individuals [2]. The cumulative risk of developing GC in Saudi Arabia is 0.31%, significantly lower than the global cumulative risk of 1.31% [3].

Adenocarcinoma is the predominant histological type of GC, accounting for 90-95% of all cases, and is categorized into diffuse and intestinal subtypes [4]. The epidemiology of GC is influenced by various etiological factors that differ across geographical regions and between developed and developing countries. In Saudi Arabia, rapid urbanization, lifestyle modifications, dietary shifts toward fast food, and exposure to risk factors like water-pipe smoking have significantly impacted GC incidence rates [5,6].

Early-stage GC is often asymptomatic or presents nonspecific symptoms indicative of ulceration or gastritis. However, as the disease progresses, symptoms such as dysphagia, weight loss, and abdominal pain become more apparent [7]. Upper gastrointestinal endoscopy remains the gold standard for diagnosis, facilitating the pathological assessment of biopsy specimens. Novel screening techniques, including electronic or virtual chromoendoscopy and magnification endoscopy, are promising to improve detection rates of precancerous gastric lesions and early-stage GC [8]. Endoscopic ultrasound is another vital modality for T staging and lymph node assessment of GC, particularly in local staging [5].

Recent advancements in diagnosis, staging, genetic stratification, surgical resection, and various treatment modalities have fostered a multidisciplinary approach to managing gastric adenocarcinoma. These include systemic chemotherapy, chemoradiation, targeted therapies, and immunotherapy [7]. Surgical resection remains crucial, aiming to excise the tumor along with surrounding tissues. At the same time, adjuvant therapies such as chemotherapy or radiotherapy may be employed to eliminate residual cancer cells and minimize the risk of recurrence [9,10].

Despite the progress made in treatment options, the prognosis for patients with GC remains dismal, primarily due to frequent delays in diagnosis until the advanced stages of the disease [9,11]. This underscores the need for enhanced screening protocols for early detection to improve patient outcomes and survival rates. The current study investigates treatment modalities for GC and survival outcomes and factors associated with mortality in Najran, Saudi Arabia.

## Materials and methods

Study design and objective

This retrospective descriptive study reviews the medical records of adults (≥ 18 years) diagnosed with GC who received treatment at King Khaled Hospital in Najran, Saudi Arabia, between January 1, 2014, and December 31, 2022.

Inclusion and exclusion criteria

Patients aged 18 years or older with a confirmed diagnosis of GC, established via histopathology, who received treatment, specifically, neoadjuvant chemotherapy followed by surgery and adjuvant chemotherapy, or surgery followed by adjuvant chemotherapy or palliative chemotherapy at our center, were included in this study. This included patients with a minimum follow-up duration of six months post-diagnosis; complete medical records available for review were also required. Patients younger than 18 years, those lacking a confirmed diagnosis of GC, patients with other synchronous and/or prior malignant tumors, or those who lost during follow-up were excluded from the study.

Data collection

The data included variables such as age at diagnosis, gender, body mass index (BMI), smoking status, and comorbidities (hypertension, diabetes mellitus, chronic renal failure, chronic liver failure, chronic obstructive pulmonary disease, hyperlipidemia), along with a family history of GC, previous *Helicobacter pylori* infection, primary symptoms, histological subtype, metastatic locations and number, and laboratory and immunohistochemistry findings (human epidermal growth factor receptor-2 (HER2), carcinoembryonic antigen (CEA), carbohydrate antigen (CA)19-9).

Information on neoadjuvant chemotherapy, adjuvant chemotherapy, palliative chemotherapy, surgery, and outcomes (survival status and recurrence) was also sourced from medical records. Treatment regimen data included the number of chemotherapy cycles, duration, and the maximum therapeutic response observed.

The cut-off values were normal CEA levels (typically < 2.5-5 ng/mL) and CA19-9 levels (usually < 37 U/mL); levels above these thresholds indicated elevation. The tumor's stage at diagnosis was established based on the criteria outlined in the 7th edition guidelines of the American Joint Committee on Cancer (AJCC). Therapeutic efficacy was evaluated using RECIST 1.1 criteria for solid tumors. Progression-free survival was defined as the time from treatment initiation to tumor progression or death from any cause. In contrast, overall survival (OS) was defined as the duration from treatment commencement until death from any cause. Intervention strategies for each patient were developed by a multidisciplinary team (MDT) convened regularly to create personalized plans. The MDT included specialists from medical and radiation oncology, pathology, radiology, and surgery. Patient evaluations were based on cancer stage and treatment tolerability. Those with localized GC (stages I to III) were assessed for surgical resection. In contrast, those with advanced metastatic disease (stage IV) received palliative chemotherapy or referrals to palliative care, as determined by the MDT.

Study outcomes

The primary outcome involved assessing the treatment modalities for GC and survival outcomes. The secondary outcome entailed identifying factors correlated with mortality in these patients.

Statistical analysis

Descriptive statistics were utilized to characterize the dataset, with quantitative variables presented as mean ± standard deviation (SD) or median (interquartile range (IQR)), depending on the normality of the data. Qualitative variables were reported as frequencies and percentages. Univariate Cox proportional hazards models were employed to assess the impact of various factors, including age, gender, smoking status, hypertension, diabetes mellitus, chronic obstructive pulmonary disease, coronary artery disease, chronic renal failure, chronic liver disease, malnutrition, stage of disease, distance metastasis, CEA, CA19-9, neoadjuvant chemotherapy, histological type, and HRE2 receptors. Statistically significant factors from the univariate analysis were then carried forward to the multivariate analysis. Associations were quantified using hazard ratios (HR) with 95% confidence intervals (CI), and a significance level of p < 0.05 was established. All analyses used SPSS Statistics version 20 (IBM Corp. Released 2011. IBM SPSS Statistics for Windows, Version 20.0. Armonk, NY: IBM Corp.).

Ethical approval

This study received approval from the Ethics Research Committee of King Khalid Hospital (code: KACST, KSA: H-11-N-081, IRB log number: 2022-258) on April 12, 2022, in accordance with the ethical principles delineated in the Declaration of Helsinki. Given the study's anonymous and retrospective nature, written informed consent from patients was not deemed necessary.

## Results

Patient characteristics, comorbidities, and laboratory findings

The mean age of patients was 64.0 ± 17.7 years (median (IQR) 64.0 (52.0, 77.0) years), with 70 individuals (57.9%) over 60 years old. The cohort comprised 86 males (71.1%) and 35 females (28.9%), resulting in a male-to-female ratio of approximately 2.5:1. Additionally, 24 patients (19.8%) were underweight (BMI < 18.6 kg/m²). The common comorbidities included hypertension in 49 patients (40.5%) and diabetes in 33 patients (27.3%). A family history of GC was noted in 12 patients (9.9%), and 16 patients (13.2%) reported current smoking. *Helicobacter pylori* infection was found in 18 patients (14.9%). The primary symptom was abdominal pain in 99 patients (81.8%), followed by weight loss and nausea in 35 patients (28.9%). HER2 receptor status was negative in 38 patients (31.4%), while elevated CEA (≥ 5 ng/ml) and CA19-9 levels (≥ 37 U/mL) were observed in 51 patients (42.1%) and 50 patients (41.3%), respectively (Table 1).

**Table 1:** Demographic and clinical profile of GC cases (N = 121) CA19-9: carbohydrate antigen 19-9, CEA: carcinoembryonic antigen, HER2: human epidermal growth factor receptor-2, IQR: interquartile range, GC: gastric cancer

Variables	N (%)
Age (years), median (IQR)	64.0 (52.0, 77.0)
Age ≥ 60 years	51 (42.1%)
Age < 60 years	70 (57.9%)
Gender	
Male	86 (71.1%)
Female	35 (28.9%)
BMI (kg/m²), mean ± SD	22.7 ± 4.6 (range: 13.0-48.9)
Underweight (< 18.6 kg/m²)	24 (19.8%)
Comorbidities	
Hypertension	49 (40.5%)
Diabetes	33 (27.3%)
Chronic obstructive pulmonary disease	16 (13.2%)
Coronary artery disease	16 (13.2%)
Chronic kidney disease	6 (5.0%)
Chronic liver failure	10 (8.3%)
Family history of GC	12 (9.9%)
Current smoking	16 (13.2%)
*Helicobacter pylori* infection	18 (14.9%)
Main symptoms	
Abdominal pain	99 (81.8%)
Weight loss	35 (28.9%)
Nausea	35 (28.9%)
Dysphagia	8 (6.6%)
Melena	6 (5.0%)
Early satiety	6 (5.0%)
Immunohistochemistry analysis	
HER2	
0; no expression of HER2 (negative)	38 (31.4%)
1+; low expression (negative)	2 (1.7%)
3+; high expression (positive)	2 (1.7%)
Not tested	79 (65.3%)
CEA (ng/ml)	
Normal (< 5 ng/ml)	70 (57.9%)
High (≥ 5 ng/ml)	51 (42.1%)
CA19-9 (U/mL)	
Normal (< 37 U/mL)	71 (58.7%)
High (≥ 37 U/mL)	50 (41.3%)

Patient treatment profiles and survival analysis

Surgical intervention was performed on 72 patients (59.5%), while 49 patients (40.5%) were considered non-resectable. Among the surgical cohort, 36 surgeries (30%) followed neoadjuvant therapy, 30 initial surgeries (25%) were performed, and six staged surgeries (5%) occurred. The resectable subgroup included 26 total gastrectomies (21.0%), 10 subtotal gastrectomies (8.3%), one gastric bypass (0.8%), three feeding tube placements (2.5%), and two endoscopic resections (1.7%). Intraoperative findings revealed 22 cases (18.2%) of non-resectable masses. Among surgical patients, 36 (30.0%) achieved R0 status, indicating clear resection margins.

The pathology findings revealed that the histological types of adenocarcinomas included intestinal type, with 95 cases (78.5%), and diffuse type, such as signet ring cell, with 26 cases (21.5%). Multiple metastases were noted in 27 patients (22.3%), primarily in the liver (n = 25, 20.7%). Tumor grades included poorly differentiated adenocarcinoma in 58 patients (47.9%), moderately differentiated in 27 (22.3%), and well-differentiated in 19 (15.7%). The most prevalent TNM stage was stage IV (n = 50, 41.3%), followed by stage III (n = 25, 20.7%) (Table 2).

**Table 2:** Treatment, histopathology, and follow-up report AJCC TNM: American Joint Committee on Cancer tumor, node, metastasis; SD: standard deviation; IQR: interquartile range

Variables	N (%)
Histopathology	
Intestinal-type	26 (21.5%)
Diffuse-type (e.g., signet ring cell)	95 (78.5%)
Disease stage	
Localized	10 (8.3%)
Locally advanced	43 (35.5%)
Metastatic	52 (43.0%)
Incomplete staging (not fit)	16 (13.2%)
Metastasis location	
Lung	7 (5.8%)
Liver	25 (20.7%)
Multiple metastases	27 (22.3%)
Cervical lymph nodes	8 (6.6%)
Bone	2 (1.7%)
Tumor grade	
Well-differentiated	19 (15.7%)
Moderately differentiated	27 (22.3%)
Poorly differentiated	58 (47.9%)
Unclassified	17 (14.0%)
Surgery	
Resectable	72 (59.5%)
Non-resectable	49 (40.5%)
Time of surgery	
After completion of neoadjuvant	36 (30%)
Upfront	30 (25%)
Interval surgery	6 (5.0%)
Seventh AJCC TNM stage	
I	13 (10.7%)
II	15 (12.4%)
III	25 (20.7%)
IV	50 (41.3%)
Incomplete	18 (14.9%)
First-line chemotherapy	
Chemotherapy	106 (87.6%)
Palliative chemotherapy	15 (12.4%)
Chemotherapy cycle number	
≤ 3	11 (9.1%)
4-6	61 (50.4%)
≥ 7	34 (28.1%)
Follow-up duration (months), mean ± SD	41.3 ± 38.8 (range 3.0-133.0)
Survival (months), median (IQR)	22 (11, 74)
Outcome	
Alive	96 (79.3%)
Died	25 (20.7%)
Recurrence	8 (6.6%)

**Figure 1:**
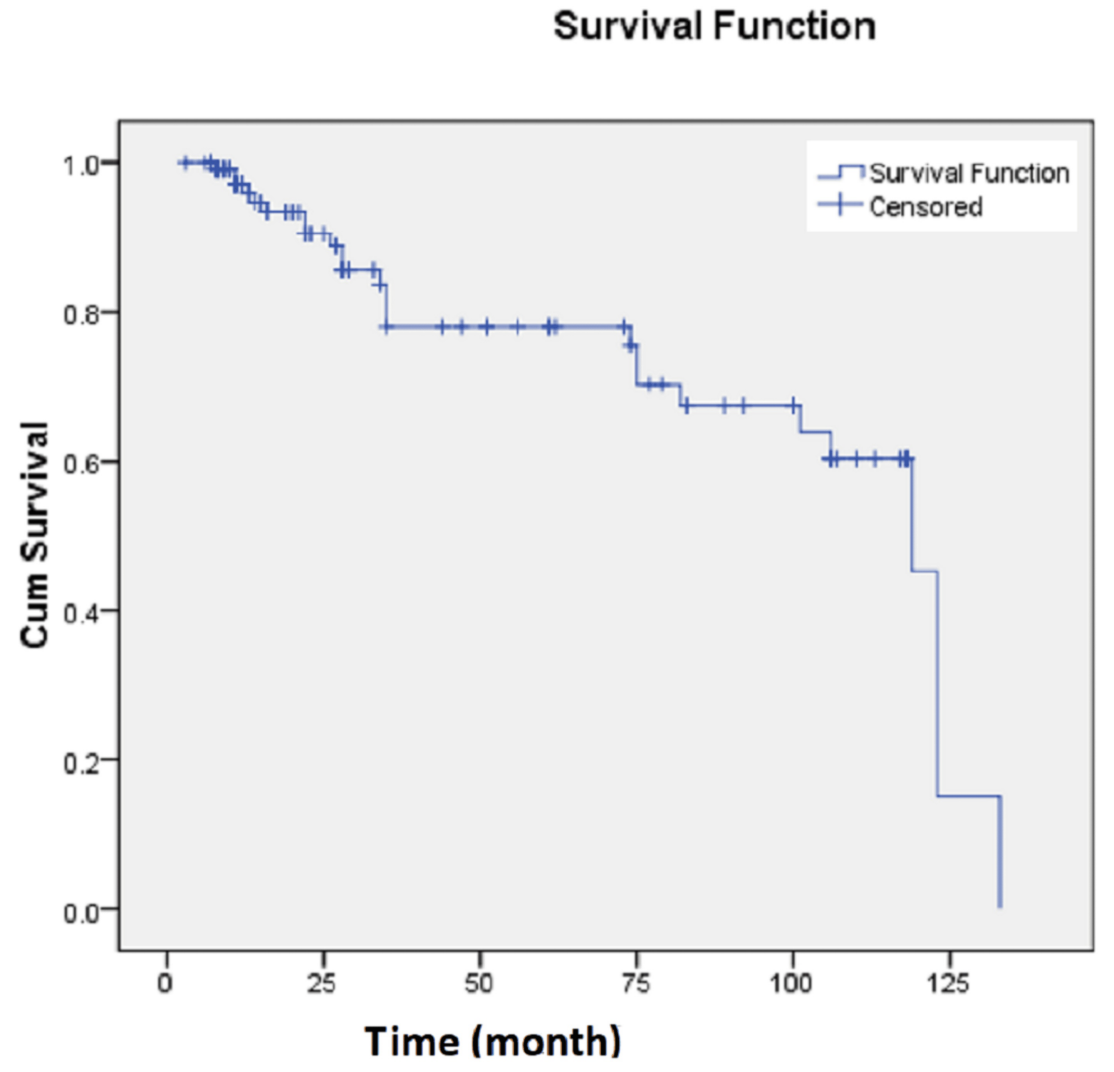
Kaplan-Meier survival curves for OS among 121 GC patients. The median OS time was 28 months (95% CI: 20-51) CI: confidence interval, GC: gastric cancer, OS: overall survival

During a follow-up period of 41.3 ± 38.8 months (range: 3.0-133.0 months), 96 patients (79.3%) were alive, while 25 patients (20.7%) had died. Relapse occurred in eight patients (6.6%). The median survival rate was 28 months (95% CI: 20-51 months) (Figure 1). Survival rates included 68.2% at 12 months (95% CI: 60.4-77.1%), 42.8% at 36 months (95% CI: 34.5-53.0%), and 37.4% at 60 months (95% CI: 29.3-47.8%).

Factors associated with mortality

In univariate analysis, factors associated with mortality in GC patients were age greater than 65 years (HR: 1.76; 95% CI: 1.17-2.65; p = 0.006), female gender (HR: 2.38; 95% CI: 1.50-3.79; p < 0.001), stage IV disease (HR: 2.29; 95% CI: 1.02-5.14; p = 0.044), high CEA levels (≥ 5 ng/mL) (HR: 1.53; 95% CI: 1.01-2.33; p = 0.047), and neoadjuvant chemotherapy (HR: 0.57; 95% CI: 0.34-0.98; p = 0.042) (Table 3).

**Table 3:** Univariate analysis of prognostic factors for mortality in GC patients p-values indicate the level of statistical significance, with a threshold of p < 0.05 considered statistically significant. HR: hazard ratio, CI: confidence interval, BMI: body mass index, NL: normal level, HRE2: human epidermal growth factor receptor-2, GC: gastric cancer, CA19-9: carbohydrate antigen 19-9

Variable	Subgroup	Total; N (%)	HR (95% CI)	p-value
Age	Age < 65 years	61 (50.4)	-	0.006
	Age > 65 years	60 (49.6)	1.76 (1.17-2.65)	
Gender	Male	86 (71.1)	-	<0.001
	Female	35 (28.9)	2.38 (1.50-3.79)	
Smoking status	Non-smoker	105 (86.8)	-	0.432
	Smoker	16 (13.2)	0.77 (0.40-1.48)	
Hypertension	No	72 (59.5)	-	0.11
	Yes	49 (40.5)	1.41 (0.93-2.15)	
Diabetes mellitus	No	88 (72.7)	-	0.644
	Yes	33 (27.3)	0.90 (0.57-1.41)	
Chronic obstructive pulmonary disease	No	105 (86.8)	-	0.711
	Yes	16 (13.2)	1.12 (0.61-2.06)	
Coronary artery disease	No	105 (86.8)	-	0.711
	Yes	16 (13.2)	1.12 (0.61-2.06)	
Chronic renal failure	No	115 (95.0)	-	0.286
	Yes	6 (5.0)	1.64 (0.66-4.06)	
Chronic liver disease	No	111 (91.7)	-	0.292
	Yes	10 (8.3)	1.48 (0.71-3.06)	
Malnutrition	Normal (BMI > 18.6 kg/m²)	97 (80.2)	-	0.416
	Malnutrition (BMI < 18.6 kg/m²)	24 (19.8)	1.24 (0.74-2.06)	
Stage of disease	Stage I	13 (10.7)	-	
	Stage II	15 (12.4)	1.43 (0.54-3.78)	0.466
	Stage III	25 (20.7)	1.46 (0.60-3.55)	0.402
	Stage IV	50 (41.3)	2.29 (1.02-5.14)	0.044
	Incomplete	18 (14.9)	2.08 (0.87-5.00)	0.101
Distance metastasis	No	52 (43.0)	-	0.727
	Yes	69 (57.0)	1.07 (0.72-1.61)	
CEA	Normal (NL: < 5 ng/mL)	70 (57.9)	-	0.047
	High (≥ 5 ng/mL)	51 (42.1)	1.53 (1.01-2.33)	
CA19-9	Normal (NL: < 37 U/mL)	71 (58.7)	-	0.85
	High (≥ 37 U/mL)	50 (41.3)	1.04 (0.69-1.56)	
Neoadjuvant chemotherapy	No	30 (41.7)	-	0.042
	Yes	42 (58.3)	0.57 (0.34-0.98)	
Histological type	Intestinal-type	95 (78.5)	-	0.75
	Diffuse-type (e.g., signet ring cell)	26 (21.5)	0.92 (0.57-1.50)	
HRE2 receptors	Negative	38 (90.5)	-	0.757
	Positive	4 (9.5)	0.80 (0.19-3.36)	

However, in multivariate analysis, age greater than 65 years showed a trend toward increased mortality, with an HR of 1.71 (95% CI: 0.99-2.95; p = 0.0520), although this association did not reach statistical significance. In contrast, the female gender demonstrated a statistically significant association with increased mortality, indicated by an HR of 3.12 (95% CI: 1.56-6.24; p = 0.0013). Additionally, stage IV disease was associated with a nearly twofold increase in mortality risk, as indicated by an HR of 1.92 (95% CI: 1.01-3.66; p = 0.0481). High CEA levels (≥ 5 ng/mL) also emerged as a significant prognostic factor, exhibiting an HR of 1.95 (95% CI: 1.08-3.53; p = 0.0277). In contrast, the administration of neoadjuvant chemotherapy was associated with a decreased mortality risk, with an HR of 0.55 (95% CI: 0.31-0.97; p = 0.0374), indicating a potential protective effect (Table 4).

**Table 4:** Multivariate analysis of prognostic factors for mortality in GC patients p-values indicate the level of statistical significance, with a threshold of p < 0.05 considered statistically significant. CI: confidence interval, GC: gastric cancer

Covariate	b	SE	Wald	P	Exp(b)	95% CI of Exp(b)
Age > 65 years	0.54	0.28	3.77	0.052	1.71	0.99 to 2.95
Female gender	1.14	0.35	10.34	0.0013	3.12	1.56 to 6.24
Stage IV	0.65	0.33	3.91	0.0481	1.92	1.01 to 3.66
High carcinoembryonic antigen (≥ 5 ng/mL)	0.67	0.3	4.85	0.0277	1.95	1.08 to 3.53
Neoadjuvant chemotherapy	-0.61	0.29	4.33	0.0374	0.55	0.31 to 0.97

**Figure 2:**
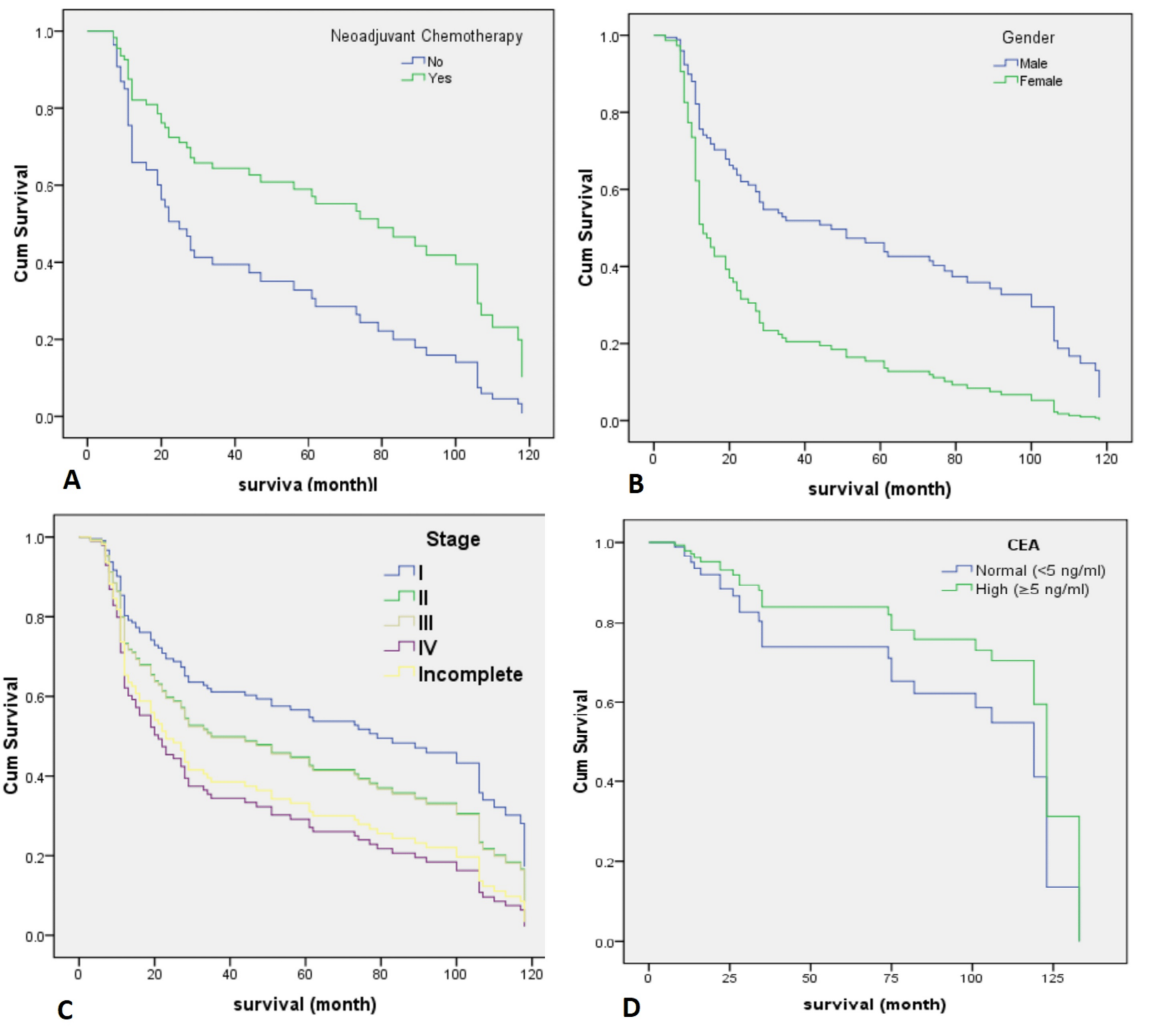
Cox regression analysis for prognostic factors impacting mortality in GC. (A) Neoadjuvant chemotherapy: HR 0.55 (95% CI: 0.31–0.97; p = 0.0374), indicating reduced mortality risk. (B) Female gender: HR 3.12 (95% CI: 1.56–6.24; p = 0.0013), indicating increased mortality risk. (C) Disease stage IV: HR 1.92 (95% CI: 1.01–3.66; p = 0.0481), indicating nearly twofold increased mortality risk. (D) Elevated CEA levels (≥ 5 ng/mL): HR 1.95 (95% CI: 1.08–3.53; p = 0.0277), indicating increased mortality risk HR: hazard ratio, CI: confidence interval, CEA: carcinoembryonic antigen, GC: gastric cancer

The survival analysis of GC patients demonstrated that neoadjuvant therapy significantly improved survival outcomes, with treated individuals exhibiting a 12-month survival rate of 85.42%, contrasted with 59.50% for those who did not receive such treatment; the survival rates for the untreated group declined to 41.46% at 36 months and further to 33.17% at 60 months (Figure 2A). Younger patients, specifically those under 65 years, showed favorable survival rates of 76.8% at 12 months, which declined to 54.7% at 36 months and 49.9% at 60 months; in contrast, patients over 65 years exhibited survival rates of 59.6% at 12 months, decreasing to 31.3% at 36 months and 25.8% at 60 months. However, it was not statistically significant in multivariate analysis. Gender disparities were also evident, with male patients reporting survival rates of 76.4% at 12 months, 48.2% at 36 months, and 43.9% at 60 months, whereas female patients showed notably lower rates of 48.1% at 12 months, with declines to 29.2% at 36 months and 19.4% at 60 months (Figure 2B). Furthermore, for stage I-III patients, the survival rates were recorded at 71.4% at 12 months, declining to 45.2% at 36 months and 39.5% at 60 months, compared to stage IV patients who had survival rates of 63.8% at 12 months, 39.2% at 36 months, and 34.3% at 60 months (Figure 2C). Additionally, the analysis of CEA levels revealed significant variations: patients with normal CEA levels (less than 5 ng/mL) had a 12-month survival rate of 66.3% (95% CI: 56.0-78.6%), which decreased to 45.9% at 36 months and 36.3% at 60 months, while those with elevated CEA levels (greater than or equal to 5 ng/mL) started with a 12-month survival rate of 70.6% (95% CI: 59.1-84.3%), but their rates declined to 38.9% at both 36 and 60 months (95% CI: 27.3-55.4%) (Figure 2D).

## Discussion

This retrospective cohort study investigated the treatment modalities, survival outcomes, and prognostic factors associated with mortality among a cohort of GC patients at a tertiary care hospital in Najran, Saudi Arabia. The calculated five-year OS rate was 37.4%. The results of this study indicated that factors such as patient age greater than 65 years, female gender, advanced disease stage, and high CEA levels were significantly associated with mortality, while neoadjuvant chemotherapy significantly improved survival.

The median age at diagnosis of GC among our patients was 64.0 years (IQR 52.0, 77.0), consistent with findings from the Middle East, Far East, and Western Hemisphere, where the median age is approximately 65 years [12-14]. We observed a decline in survival rates for individuals over the age of 65 years, and this was statistically significant in univariate analysis but not in multivariate analysis. These results align with prior research indicating reduced survival rates in older patients [15-18]. Additionally, in this report, the male-to-female ratio was 2.46:1, consistent with the demographics observed in our patient population and comparable to other global studies, where the ratio typically approximates 1.8:1 [19,20]. The higher incidence of GC in males compared to females can be attributed to a combination of genetic, environmental, and behavioral factors, including alcohol consumption, smoking, differences in healthcare-seeking behaviors, and variations in the utilization of healthcare resources. Our findings indicate that female patients exhibited higher mortality rates than their male counterparts, and this difference achieved statistical significance in the multivariate analysis. Consistent with our results, other reports show lower survival rates for female patients with GC [15,21]. Conversely, other studies have statistically confirmed the significant influence of gender on improved survival outcomes among females [16,17].

In the current research endeavor, eight patients (6.6%) diagnosed with GC experienced a recurrence; however, their survival rates did not significantly differ from those of other patients in the cohort. Furthermore, 52 patients (43.0%) developed metastases, and their survival rates were markedly lower than those of the remaining patients. These findings are consistently supported by previous studies [15,18,22,23]. The predominant sites of metastasis included multiple anatomical regions, liver involvement, and lymph node dissemination. Nonetheless, no statistically significant differences in patient survival rates were observed based on the metastatic site. This outcome was anticipated because these individuals were classified as stage IV. A similar conclusion was reported by Zare et al. [15].

The guidelines propose chemotherapy as the primary therapeutic approach for advanced GC [24]. For individuals diagnosed with stage II or III GC, the guidelines advocate for tumor resection accompanied by D2 lymphadenectomy and subsequent adjuvant chemotherapy. In cases of resectable GC classified as stage IV, the recommendation is to pursue perioperative chemotherapy [18,24]. Our research indicated that patients with early-stage GC exhibited diminished mortality rates, potentially attributable to superior curative treatment alternatives, despite the persistently elevated five-year mortality rates diminishing at a gradual pace. Consequently, it is imperative to augment monitoring efforts to enhance the prognosis and amplify investments to discover more effective treatment modalities. Li et al. reported a comparable finding [18]. Furthermore, in this study, neoadjuvant chemotherapy was correlated with improved survival outcomes compared to patients with GC who did not receive such treatment and those with non-resectable disease. Our findings are consistent with previous literature on this subject [25,26]. In a study by Xu et al., the authors reported that neoadjuvant chemotherapy can enhance the prognosis for patients with large type 3 GC. However, it is noteworthy that neoadjuvant chemotherapy did not confer significant survival benefits for patients with type 4 GC [25].

Our study establishes a five-year OS rate of 37.4% for GC, consistent with the existing literature and emphasizing the considerable variability in disease outcomes attributable to disease stage and treatment modalities. This finding is corroborated by the research conducted by Wu et al., who reported a five-year OS rate of 25.83%, and Alshahrani et al., whose study indicated a survival rate of 19.6% [10,27]. Notably, stage I patients exhibited superior one-, three-, and five-year survival rates of 76.15%, 56.41%, and 56.41%, respectively, while stage IV patients faced significantly poorer outcomes of 63.82%, 39.24%, and 34.33%, respectively. These findings support the comprehensive review of prognostic factors by Mantziari et al., which posits that advanced-stage patients were subject to unfavorable outcomes due to tumor microenvironment and treatment heterogeneity [13]. Interestingly, in regions with high GC rates, such as Japan and Korea, the five-year survival rates exceed 70% [28]. However, many patients were diagnosed at advanced stages, where standard treatments like surgery, chemotherapy, or radiation often fail to improve survival outcomes. Consequently, GC patients face low survival rates following surgery [29]. Our findings highlight the importance of early detection and intervention, emphasizing the need for better diagnostic tools and personalized treatment approaches to improve patient outcomes.

CEA and CA19-9 are standard biomarkers of GC. In our study, elevated CEA levels were statistically significantly correlated with poorer survival outcomes. The findings were congruent with previous studies demonstrating that increased CEA levels resulted in significantly worse survival rates than CEA-negative individuals [30-32]. In this study, HER2-positive individuals were not statistically significantly associated with diminished survival. Recent literature has indicated that patients with HER2-positive advanced GC may experience a more favorable prognosis than their HER2-negative counterparts, particularly when treated with trastuzumab [33,34].

Perioperative chemotherapy has established itself as the standard treatment for resectable localized GC, significantly improving survival rates compared to surgery alone, as demonstrated in clinical trials like MAGIC, which evaluated regimens such as epirubicin, cisplatin, and fluorouracil or FLOT (fluorouracil, leucovorin, oxaliplatin, and docetaxel) [35]. Our findings corroborate this literature, indicating that neoadjuvant chemotherapy is associated with enhanced survival and superior outcomes, as supported by trials such as PRODIGY in Korea and RESOLVE in China, which highlighted the benefits of neoadjuvant approaches like SOX (S-1 plus oxaliplatin) [36,37]. Furthermore, adjuvant chemotherapy continues to be recommended for stage II or III disease following surgical intervention, with significant survival advantages noted in trials like CLASSIC and ACTS-GC for CAPOX and S-1 monotherapy; however, for patients with microsatellite instability-high, it appears that adjuvant chemotherapy may offer limited benefit, leading to updated guidelines favoring observation [38]. The role of adjuvant chemoradiotherapy (CRT) remains controversial. At the same time, some studies from North America suggest a survival advantage. More recent trials like ARTIST indicate only marginal benefits, particularly for patients with extensive lymphadenectomy, prompting current recommendations against adjuvant CRT in such cases [39]. Our findings align with existing evidence, affirming that neoadjuvant chemotherapy is critical in managing resectable GC, reinforcing the effectiveness of established regimens and their importance in clinical practice.

Study limitations

The present study is constrained by its retrospective monocentric design and low sample size. It evaluated the electronic medical records of patients diagnosed with GC who received treatment at the King Khaled Hospital in Najran, Saudi Arabia. Additionally, due to the nature of this single-center research, it cannot eliminate potential selection biases. A retrospective analysis of patients' definitive diagnoses may not necessarily correlate with their primary complaints, necessitating a more comprehensive and sensitive approach utilizing cancer-related diagnostic instruments. Future prospective investigations employing a systematic registry of consecutive cases with extended follow-up periods are essential to substantiate our findings.

## Conclusions

The current study's observed five-year survival rate was 37.4% (95% CI: 29.3-47.8%). This study highlights that female gender, advanced disease stage, and elevated CEA levels are significant predictors of increased mortality in GC patients. Conversely, neoadjuvant chemotherapy may lower the risk of mortality. These findings underscore the importance of personalized treatment plans and comprehensive risk assessments to enhance patient outcomes in high-risk cases. However, further research is necessary to determine whether other factors influence the selection of the most effective treatment.
